# A psychometric evaluation of Chinese chronic hepatitis B virus infection-related stigma scale using classical test theory and item response theory

**DOI:** 10.3389/fpsyg.2023.1035071

**Published:** 2023-02-01

**Authors:** Sirui Zhong, Yuxiao Zhou, Wuerken Zhumajiang, Lifen Feng, Jing Gu, Xiao Lin, Yuantao Hao

**Affiliations:** ^1^School of Public Health, Sun Yat-sen University, Guangzhou, China; ^2^Department of Disease Control and Prevention, Putian Municipal Health Commission, Putian, China; ^3^Guangdong Health Commission Affairs Center (External Health Cooperation Service Center of Guangdong Province), Guangzhou, China; ^4^Peking University Center for Public Health and Epidemic Preparedness and Response, Beijing, China

**Keywords:** chronic hepatitis B virus infection, stigma, classical test theory, item response theory, validation

## Abstract

**Purpose:**

To validate the hepatitis B virus infection-related stigma scale (HBVISS) using Classical Test Theory and Item Response Theory in a sample of Chinese chronic HBV carriers.

**Methods:**

Feasibility, internal consistency reliability, split-half reliability and construct validity were evaluated using a cross-sectional validation study (*n* = 1,058) in Classical Test Theory. Content validity was assessed by COnsensus-based Standards for the selection of health Measurement INstruments (COSMIN) criteria. The Item Response Theory (IRT) model parameters were estimated using Samejima’s graded response model, after which item response category characteristic curves were drawn. Item information, test information, and IRT-based marginal reliability were calculated. Measurement invariance was assessed using differential item functioning (DIF). SPSS and R software were used for the analysis.

**Results:**

The response rate reached 96.4% and the scale was completed in an average time of 5 min. Content validity of HBVISS was sufficient (+) and the quality of the evidence was high according to COSMIN criteria. Confirmatory factor analysis showed acceptable goodness-of-fit (*χ*^2^/df = 5.40, standardized root mean square residual = 0.057, root mean square error of approximation = 0.064, goodness-of-fit index = 0.902, comparative fit index = 0.925, incremental fit index = 0.926, and Tucker-Lewis index = 0.912). Cronbach’s α fell in the range of 0.79–0.89 for each dimension and 0.93 for the total scale. Split-half reliability was 0.96. IRT discrimination parameters were estimated to range between 0.959 and 2.333, and the threshold parameters were in the range-3.767 to 3.894. The average score for test information was 12.75 (information >10) when the theta level reached between-4 and + 4. The IRT-based marginal reliability was 0.95 for the total scale and fell in the range of 0.83–0.91 for each dimension. No measurement invariance was detected (d-*R*^2^ < 0.02).

**Conclusion:**

HBVISS exhibited good feasibility, reliability, validity, and item quality, making it suitable for assessing chronic Hepatitis B virus infection-related stigma.

## 1. Introduction

Hepatitis B virus (HBV) infection is a significant global public health problem. In 2019, the World Health Organization estimated a global prevalence of HBV infection in the general population of 3.8%, amounting to approximately 296 million people living with chronic hepatitis B virus infection (WHO, 2021). HBV causes high mortality rates due to complications such as cirrhosis, hepatic decompensation, and hepatocellular carcinoma. Despite remarkable changes in the healthcare and hepatitis vaccination programs recently rolled out in China, the country still bears the highest disease burden in the world, with over one-third of the world’s HBV infections and deaths occurring in China (Chen et al., 2018). This heavy infection burden is accompanied by the stigma associated with chronic HBV infection (Wallace et al., 2017; Mokaya et al., 2018; Le et al., 2019; Zhu et al., 2019; Smith-Palmer et al., 2020). In China, evidence of stigma that marginalizes chronic HBV carriers from diverse social aspects is conclusive (Liu, 2016; Wallace et al., 2017; Han et al., 2018). Separation, manifested as social exclusion, is the most common form of stigma, followed by status loss and discrimination (Jin et al., 2021). System reviews have also shown that HBV-related stigma is a barrier to healthcare for HBV infection including prevention, diagnosis, and treatment strategies, and has potential negative impacts on mental health and well-being (Ellard and Wallace, 2013; Vedio et al., 2017; Mokaya et al., 2018). HBV-related stigma is mostly driven by fear of contagion due to a lack of knowledge of transmission routes (Lee et al., 2016; Jin et al., 2021) as well as negative preconception that those who become infected with HBV in adults were attributed to substance misuse and sexual promiscuity (Smith-Palmer et al., 2020). Despite a much greater global prevalence and longer course of disease (Bartenschlager et al., 2018), studies on conceptualizing HBV stigma are much lesser than HCV, which can be theorized as identity, embodiment, institutionalization, and structuration (Harris et al., 2021). Moreover, HBV-related stigma has been less administrated than HIV, which may partly attribute to a lack of political prioritization around HBV compared with the substantial public health interventions on HIV (Smith-Palmer et al., 2020).

Development and validation of comprehensive and objective measurement of the stigma related to chronic HBV infection is a prerequisite for implementing appropriate interventions to eliminate and reduce stigma. However, studies on measuring HBV-related stigma in China are limited. Most previously purposed HBV-related stigma measurements in China are based solely on perception or knowledge scales that quantifies broad negative emotions in the general population toward HBV-positive individuals and few studies have focused on measuring stigma from the perspective of chronic HBV infection patients (Jin et al., 2021). To fill this gap, the hepatitis B virus infection-related stigma scale (HBVISS) has been developed through the standard scale-development procedures by our research group (Lifen et al., 2011) and initially validated in a 151 sample using Classical Test Theory (CTT) analysis in 2012 (Lifen et al., 2012). Our HBVISS is a self-reported scale designed to assess the stigma that hepatitis B-infected patients perceive in their everyday lives. In the process of scale development, it took into account not only the opinions of doctors and experts but also the consideration of patients and their families, making it more in line with the actual feelings of patients. Although the scale has shown good psychometric properties in the preliminary validation (Lifen et al., 2012), the scale still needs to be verified comprehensively and scientifically *via* a larger representative sample for better application in real-world study. CTT is a traditional quantitative approach to testing the reliability and validity of a scale based on the observed score, while Item Response Theory (IRT) attempts to explain the connection between observed item responses on scales and subjects’ latent traits based on item characteristic curves (Cappelleri et al., 2014). Moreover, IRT provides a unique advantage of sample independence of estimated parameters, which would enhance the extrapolation of the analysis results (DeVellis, 2006; Thomas, 2019). Therefore, a combination of CTT and IRT analysis allows us to have a deeper understanding of the psychometric properties of HBVISS.

Scale validation is a crucial step before its general application. However, the evidence to support the psychometric properties of identified HBV-related stigma instruments is very limited currently (Smith-Palmer et al., 2020). This article aims to further validate the psychometric properties of our HBVISS by implementing the CTT and IRT analysis, in order to quantitatively examine its suitability for the assessment of chronic HBV infection-related stigma, with the ultimate goal to better measure HBV-related stigma in China.

## 2. Materials and methods

### 2.1. Instruments

Hepatitis B virus infection-related stigma scale consists of 23 items, which can be concluded in 5 dimensions including received stigma, negative self-perception, perceived stigma, disease secrecy and secondary stigma. A 5-point Likert scale was used as the item response format. The options of all items were 1 = strongly disagree, 2 = disagree, 3 = neutral, 4 = agree, and 5 = strongly agree, with higher scores indicating stronger levels of chronic HBV infection-related stigma. The construct and content of HBVISS are shown in Table 1.

**Table 1:** The dimensions and items of hepatitis B virus infection-related stigma scale.

Dimension	Item code	Item
Received stigma	R1	I once suffered from unfair treatment because of the hepatitis B infection (rejected from admission, work, dating, marriage, sex, etc.).
	R2	My family was reluctant to talk to others about hepatitis B infection for fear of repulsion.
	R3	Because of my hepatitis B infection, I had been pushed aside by my friends.
	R4	Because of my hepatitis B infection, I was refused to be cared for or to contact his/her child.
	R5	Because of my hepatitis B, I feel like myself being treated differently.
Negative self-perception	N6	I feel unlucky and unfair being infected with hepatitis B.
	N7	After being infected with hepatitis B, I felt like a burden on my family/society.
	N8	People’s attitude toward someone with HBV infection make me feel worse about myself.
	N9	I worry about being disliked and avoided, so I do not initiate interactions with others.
Perceived stigma	P10	Because of my hepatitis B infection, I think people around will talk about me behind my back.
	P11	Because of my hepatitis B infection, I feel some people feel uncomfortable around me.
	P12	Because of my hepatitis B, I will feel embarrassed by what people thought of me.
	P13	I think others will treat the hepatitis B infection differently.
	P14	I do not think other people want to make friends with hepatitis B infection.
	P15	I think hepatitis B would make infected people not able to enjoy the same rights as ordinary people.
	P16	I think hepatitis B will cost infected people their jobs.
Disease secrecy	D17	I do not want to tell others about my hepatitis B infection.
	D18	I will only tell the people I trust about my hepatitis B infection.
	D19	I want those who know about my hepatitis B infection to keep me a secret.
	D20	I am afraid that going to the hospital would let people know about my hepatitis B infection.
Secondary stigma	S21	Because of my hepatitis B infection, my relatives and friends visit me less frequently than before.
	S22	Because of my hepatitis B infection, my family feels shamed.
	S23	Because of my hepatitis B infection, my family is repelled by the others.

### 2.2. Participants

One thousand ninety-seven participants were recruited in two first-class general hospitals in Guangdong Province, China --The Third Affiliated Hospital of Sun Yat-sen University and the Eighth People’s Hospital of Guangzhou. A convenience sampling method was used. Participants from both the outpatient and inpatient departments of these hospitals were recruited if they met all of the following inclusion criteria:

1. People with chronic HBV infection, including asymptomatic chronic HBV carriers and patients with chronic hepatitis B (CHB) and/or hepatitis B-induced liver cirrhosis. Participants must have had a diagnosed chronic HBV infection for 1 year or longer by doctors, following the guidelines for the prevention and treatment of CHB (2010 version) (Chinese Society of Hepatology and Chinese Society of Infectious Diseases Chinese Medical Association, 2011).
2. People aged 18–60 years who were able to read, write, and communicate with basic understanding capabilities.
3. People with the ability to complete the survey on their own or with the help of investigators.
4. People who volunteered for participation and were willing to sign an informed consent form.

The exclusion criteria were as follows:

1. People with liver cancer, pulmonary or neurological diseases, or co-infection with other hepatitis viruses or HIV.
2. People with disabilities, past or present chronic infectious diseases, or mental disorders.
3. People who had been critically ill.

### 2.3. Data collection

Participants were asked to independently complete a questionnaire composed of two parts, one of which was an assessment of self-reported socio-demographic information on gender, age, residence, education, marital status, diagnosis, *per capita* annual disposable income, course of illness, and treatment status; the other part was HBVISS. In HBVISS, the sum of all answers to items within a given dimension represented the dimension score, which along with the total score, was transformed into a 0–100 scale to allow better interpretation and comparison.

### 2.4. Statistical analysis

#### 2.4.1. Feasibility and descriptive statistics

The scale feasibility was determined by the response rate and average time to complete the scale. The mean, standard deviation (SD), skewness, kurtosis, missing number and rate, and percentage of responses at the floor and ceiling of each item were described. Generally, a highest score/lowest score ratio of >25% indicated the presence of a ceiling/floor effect (Raat et al., 2007). The average total score and dimension scores were also calculated.

#### 2.4.2. COnsensus-based standards for the selection of health measurement INstruments analysis for content validity

COSMIN (COnsensus-based Standards for the selection of health Measurement INstruments), which includes a consensus-based methodology for rating the content validity of PROMs (Patient-Reported Outcome Measures) (Terwee et al., 2018a), was used. The COSMIN methodology for evaluating the content validity of PROMs consists of three steps (Terwee et al., 2018b). Firstly, we used qualitative measurement to score each step of the scale development. Secondly, we evaluated the content validity by asking patients and professionals about the relevance, comprehensiveness, and comprehensibility of the PROM. Thirdly, we evaluated the content validity of the PROM, based on the quality and results of the available studies and the PROM itself, using the scoring system (see details in www.cosmin.nl). Data acquisition and score assignments were conducted by two graduate students majoring in Medical Statistics at Sun Yat-sen University.

#### 2.4.3. Classical test theory analysis

The scale’s internal consistency reliability was assessed by calculating Cronbach’s *α* α coefficient, and generally, *α* > 0.70 is acceptable (Charter, 2003). Split-half reliability was assessed by calculating the correlation of two halves of the scale, which were split based on odd-numbered and even-numbered items (Uloku, 2022). The construct validity of the theoretical 5-factor model was assessed by confirmatory factor analysis (CFA). The standardized path diagram was drawn to depict the relationship between the factors and items. The model fit was examined using the ratio *χ*^2^/degree of freedom (*χ*^2^/*df*), root mean square error of approximation (RMSEA), standardized root mean square residual (SRMR), goodness-of-fit index (GFI), comparative fit index (CFI), incremental fit index (IFI), and Tucker-Lewis index (TLI). A *χ*^2^/*df* ratio ≤ 5.00 demonstrated an acceptable model fit (Gil-Monte et al., 2010). The fit was considered to be adequate when the RMSEA and SRMR were ≤ 0.08, and the GFI, CFI, IFI, and TLI were ≥ 0.90 (Hu and Bentler, 1999).

#### 2.4.4. Item response theory analysis

We first tested the two basic assumptions of IRT: unidimensionality and local independence. We used exploratory factor analysis (EFA) to test the unidimensionality. It would be considered a convincing indicator of unidimensionality if the ratio of the eigenvalues of the first and second unrotated EFA components was greater than 3:1 (Ye et al., 2018). Local independence was examined by the residual correlation matrix produced by the single factor CFA, and items with residual correlations <0.20 were deemed to be acceptable (Reeve et al., 2007).

Upon satisfaction of the two assumptions, we fitted Samejima’s two-parameter graded response model (Samejima, 1997), which is specifically an extension of the 2-Parameter Logistic Model for ordered polytomous items (Hambleton et al., 2010). According to Samejima’s theory, the distinguishing power of a multilevel item remains the same, but the threshold parameters increase as the score increases (Embretson and Reise, 2000). Therefore, we obtained one discrimination parameter (*a*) and four threshold parameters (*b_i_*) for each item. Larger values of discrimination parameters implied that the related items were more capable of identifying individuals at different trait levels, and threshold parameters should monotonically increase with the category (Kamudoni et al., 2018). Item response category characteristic curves were drawn. Response categories function optimally when each category has a distinct peak on the category probability curve, and the peaks are dispersed across all levels of the latent trait (Hendriks et al., 2012; Kamudoni et al., 2018).

In IRT, precision is conceptualized as information of each item. The more information obtained by the item for a given trait level, the more precise and reliable the prediction (Chiesi et al., 2018). The item information varies with the latent trait level being measured. We drew item information curves and test information curves, to show the amount of information on each item of five dimensions and the cumulative information of the scale given the various latent trait, respectively. IRT-based marginal reliability was also evaluated. The marginal reliability, an IRT-based analog to α, is an average of the reliability over different trait levels and accurately characterizes the measurement precision. As shown by a previous study, marginal reliability of 0.623 is generally acceptable (Zakria et al., 2019).

In addition, we evaluated differential item functioning (DIF) to explore whether people from different groups with equal abilities had unequal item response probabilities. The presence, magnitude, and impact of DIF concerning gender (male and female), age (18–35 and 36–60 years old), case source (hospitalized, outpatient), residence (urban and rural area), marital status (married and unmarried), education (junior middle school and below, senior high school and above), occupation (manual worker, mental worker, unemployed), income (average monthly family income < ¥1,499, ¥1,500–3,499, and > ¥3,500), and diagnosis (asymptomatic HBV carriers, CHB patients, and hepatitis B-induced cirrhosis patients) were evaluated using ordinal logistic regression models (Crane et al., 2006). A ≤ 0.02 change in McFadden’s pseudo R^2^ between the models was used as a criterion for the absence of DIF (Choi et al., 2011).

#### 2.4.5. Computation tools

The collected data were analyzed using SPSS/Win 26.0 (IBM Corp., Armonk, NY, United States). CFA was analyzed using SPSS Amos 26. IRT analyzes were performed in the RStudio environment for R (version 4.1.2). The R package ltm (version 1.2–0), mirt (version 1.36.1) and lordif (version 0.3–3) were used.

#### 2.4.6. Ethics approval and consent to participate

All procedures performed in studies were approved by the Ethics Committee of School of Public Health, Sun Yat-sen University. Informed consent was obtained from all individual participants included in the study.

## 3. Results

### 3.1. Basic characteristics of the participants

One thousand fifty-eight participants were finally included in the study. Table 2 lists a descriptive analysis of the participants’ demographic characteristics. Most of the participants (81.0%) were male. Nearly half were from urban areas, and the other half were from rural areas. Most of the participants were married, employed, and had at least a senior high school diploma (Table 2).

**Table 2:** Participants’ demographic and disease characteristics (*n* = 1,058).

Demographic characteristics	*N^a^*	%
Gender		
Male	857	81.0
Female	201	19.0
Age		
18–35	651	61.5
36–60	407	38.5
Case source		
Hospitalized	241	22.8
Outpatient	817	77.2
Residence		
Urban area	513	48.5
Rural area	545	51.5
Marital status		
Married	749	70.8
Unmarried	309	29.2
Education		
Junior middle school and below	289	27.3
Senior high school and above	769	72.7
Occupational classification		
Manual worker	480	45.4
Mental worker	510	48.2
Unemployed	68	6.4
Average monthly family income (¥)		
≤1,499	278	26.3
1,500–3,499	480	45.4
≥3,500	294	27.8
Diagnosis		
Asymptomatic HBV carriers	77	7.3
CHB patients	819	77.4
Hepatitis B-induced cirrhosis patients	143	13.5
Self-rated illness		
Mild	623	58.9
Moderate	349	33.0
Severe	72	6.8

^a^Sample sizes within characteristics may not sum to *n* = 1,058 due to missing values. HBV, hepatitis B virus; CHB, chronic hepatitis B.

### 3.2. Feasibility and descriptive statistics

We distributed 1,097 questionnaires at the study site and 1,058 of them were returned; the response rate was 96.4%. The questionnaire was completed in an average time of 5 min. The means of item scores ranged from 2.17 to 3.62, the standard deviation ranged from 0.83 to 1.20, the skewness coefficient was-0.77–0.86, and item kurtosis was-0.99–1.23. The range of percentages of response at the floor (score = 1) was 3.02–33.27% and that at the ceiling (score = 5) was 1.52–17.11% (Table 3). The average total score of HBVISS was 45.62, and the average dimension scores ranged from 31.84 to 59.92 (range 0–100), with the highest score for disease secrecy and the lowest score for secondary stigma (Table 4).

**Table 3:** Descriptive statistics of hepatitis B virus infection-related stigma scale (HBVISS) items (*n* = 1,058).

Item code	Mean ± SD	Skewness	Kurtosis	Floor (%)	Ceiling (%)	Missing number	Missing rate (%)
							
R1	2.24 ± 1.10	0.45	−0.67	33.27	2.75	3	0.28
R2	3.23 ± 1.04	−0.21	−0.54	5.39	10.40	0	0
R3	2.67 ± 0.99	0.21	−0.48	10.84	3.14	6	0.57
R4	2.79 ± 1.02	0.17	−0.57	9.23	4.76	7	0.66
R5	2.79 ± 1.02	0.26	−0.48	8.18	5.42	7	0.66
N6	2.78 ± 1.20	0.20	−0.99	15.07	8.53	3	0.28
N7	3.10 ± 1.17	−0.10	−0.96	9.07	11.34	0	0
N8	2.92 ± 1.05	0.14	−0.69	7.20	6.82	2	0.19
N9	2.47 ± 0.98	0.54	−0.14	13.43	3.02	1	0.09
P10	2.61 ± 0.95	0.38	−0.29	9.46	2.93	1	0.09
P11	2.65 ± 0.95	0.35	−0.42	8.61	2.74	1	0.09
P12	2.83 ± 1.00	0.19	−0.64	7.20	4.45	3	0.28
P13	3.14 ± 1.05	−0.17	−0.77	5.48	7.84	0	0
P14	2.82 ± 1.01	0.22	−0.58	7.48	4.83	2	0.19
P15	3.10 ± 1.10	−0.05	−0.94	5.96	9.27	1	0.09
P16	3.10 ± 1.13	0.00	−0.96	6.62	11.25	0	0
D17	3.58 ± 1.04	−0.60	−0.28	3.59	17.11	0	0
D18	3.62 ± 0.99	−0.77	0.06	3.02	14.56	0	0
D19	3.56 ± 1.02	−0.53	−0.30	3.04	16.13	4	0.38
D20	2.83 ± 1.07	0.31	−0.72	7.68	7.11	3	0.28
S21	2.38 ± 0.89	0.69	0.46	12.13	2.18	3	0.28
S22	2.27 ± 0.87	0.83	0.96	15.09	2.28	4	0.38
S23	2.17 ± 0.83	0.86	1.23	17.44	1.52	3	0.28

SD, Standard deviation.

**Table 4:** Scale descriptive statistics and reliability assessment.

Dimension (number of items)	Mean ± SD	Cronbach’s *α* coefficient
Received stigma (5)	43.68 ± 19.09	0.79
Negative self-perception (4)	45.45 ± 21.66	0.79
Perceived stigma (7)	47.33 ± 19.95	0.89
Disease secrecy (4)	59.92 ± 20.78	0.82
Secondary stigma (3)	31.84 ± 19.10	0.86
Total scale (23)	45.62 ± 15.77	0.93

### 3.3. Classical test theory analyzes and COSMIN results

#### 3.3.1. Validity

**Figure 1:**
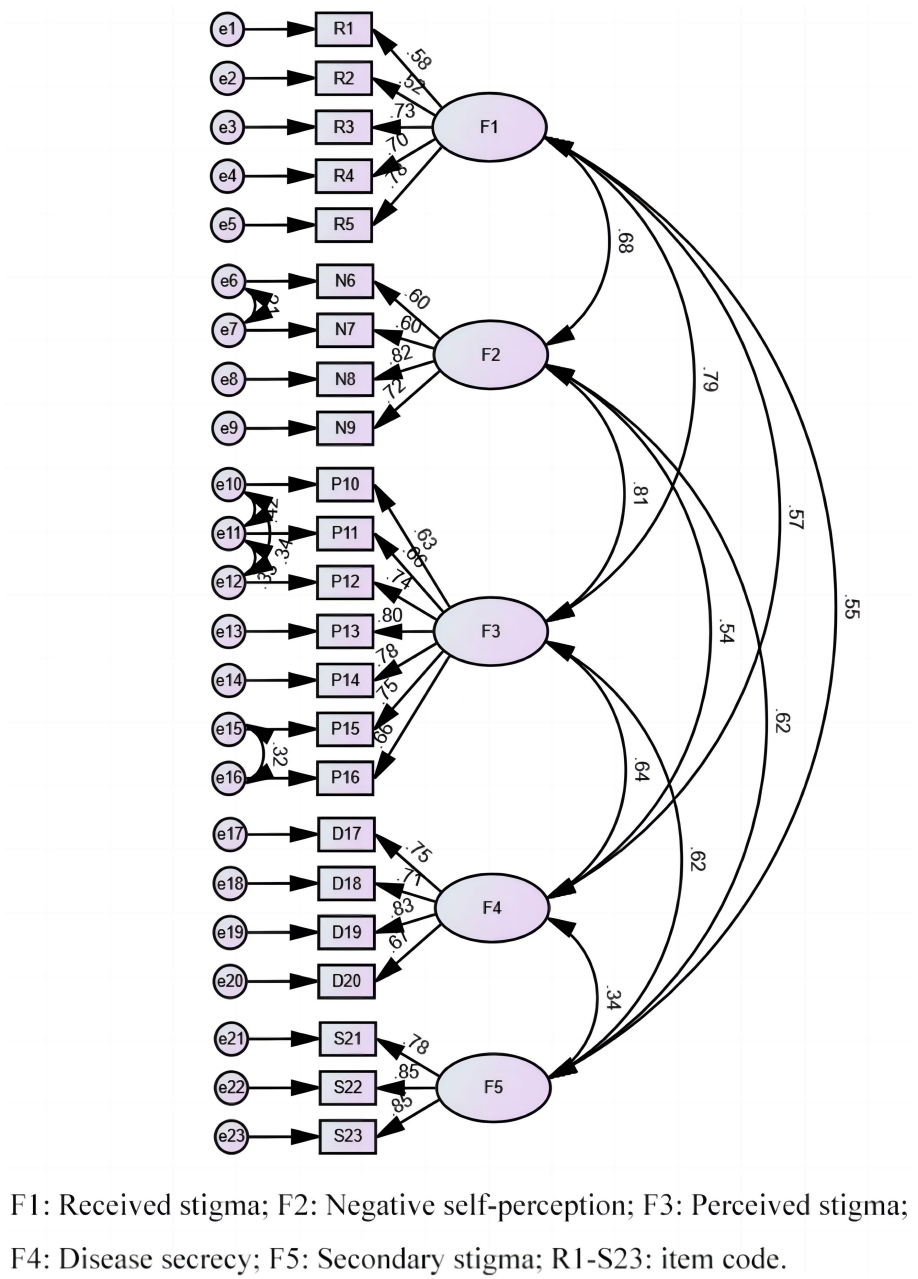
Standardized path diagram of confirmatory factor model.

According to the scoring standards of COSMIN and the actual scale development process, we finally concluded that the overall content validity of HBVISS was sufficient (+) and the quality of the evidence was high. The result of the final step is presented in Appendix 1. The CFA exhibited adequate fit (*χ*^2^/*df* = 5.40, SRMR = 0.057, RMSEA = 0.064, GFI = 0.902, CFI = 0.925, IFI = 0.926, and TLI were = 0.912) and strong item factor loadings [ranging from 0.52 to 0.85, all >0.5 and most >0.7 (Figure 1)] (Korn et al., 2021), implying that HBVISS had acceptable construct validity.

#### 3.3.2. Reliability

Cronbach’s *α* coefficient was 0.79–0.89 for each dimension and 0.93 for the total scale, which was above the threshold of 0.70 (Table 4). The correlation of the two halves of the scale was 0.96, showing great split-half reliability.

### 3.4. Item response theory analyzes results

#### 3.4.1. Unidimensionality and local independence

The ratio of the eigenvalues of the first and second unrotated EFA components was 4.32, confirming the unidimensionality of HBVISS. The residual correlation matrix produced by a single factor CFA showed that out of 253 unique item pairs, only 10 pairs (4.0%) had a residual correlation of ≥0.20, thereby fulfilling the requirement of local independence.

#### 3.4.2. Discrimination and threshold parameters

Table 5 shows the values of the discrimination (*a*) and threshold parameters (*b*_1,_ *b*_2,_ *b*_3,_ and *b*_4_) for the 23 items in HBVISS. The estimated IRT discrimination parameters ranged from 0.959 to 2.333 and the threshold parameters ranged from-3.767 to 3.894. All item discrimination values were > 0.9, indicating moderate to very high discriminatory power (Baker, 1985). The threshold parameters were also evenly spaced, indicating that the item categories were appropriately separated in terms of latent trait measurement.

**Table 5:** Estimated item response theory (IRT) parameters of hepatitis B virus infection-related stigma scale (HBVISS) (*n* = 1,058).

Item code	*a*	*b*_1_	*b*_2_	*b*_3_	*b*_4_
R1	1.097	−0.865	0.259	2.072	3.766
R2	0.959	−3.475	−1.459	0.287	2.560
R3	1.446	−2.010	−0.236	1.210	3.045
R4	1.334	−2.259	−0.379	1.039	2.796
R5	1.836	−2.008	−0.318	0.912	2.244
N6	1.176	−1.885	−0.198	0.759	2.437
N7	1.204	−2.399	−0.704	0.313	2.096
N8	2.078	−1.982	−0.440	0.589	1.957
N9	1.587	−1.705	0.224	1.389	2.865
P10	1.868	−1.856	−0.022	1.191	2.681
P11	1.930	−1.927	−0.076	1.072	2.699
P12	2.333	−1.916	−0.312	0.698	2.155
P13	2.252	−2.133	−0.664	0.219	1.783
P14	2.138	−1.940	−0.320	0.756	2.174
P15	2.084	−2.145	−0.529	0.261	1.713
P16	1.709	−2.227	−0.548	0.302	1.696
D17	1.186	−3.396	−1.643	−0.543	1.645
D18	1.096	−3.767	−1.810	−0.825	1.899
D19	1.259	−3.446	−1.627	−0.426	1.632
D20	1.350	−2.409	−0.271	0.821	2.383
S21	1.442	−1.909	0.458	1.897	3.354
S22	1.449	−1.678	0.672	2.101	3.303
S23	1.315	−1.607	0.949	2.503	3.894

#### 3.4.3. Item response category characteristic curves

**Figure 2:**
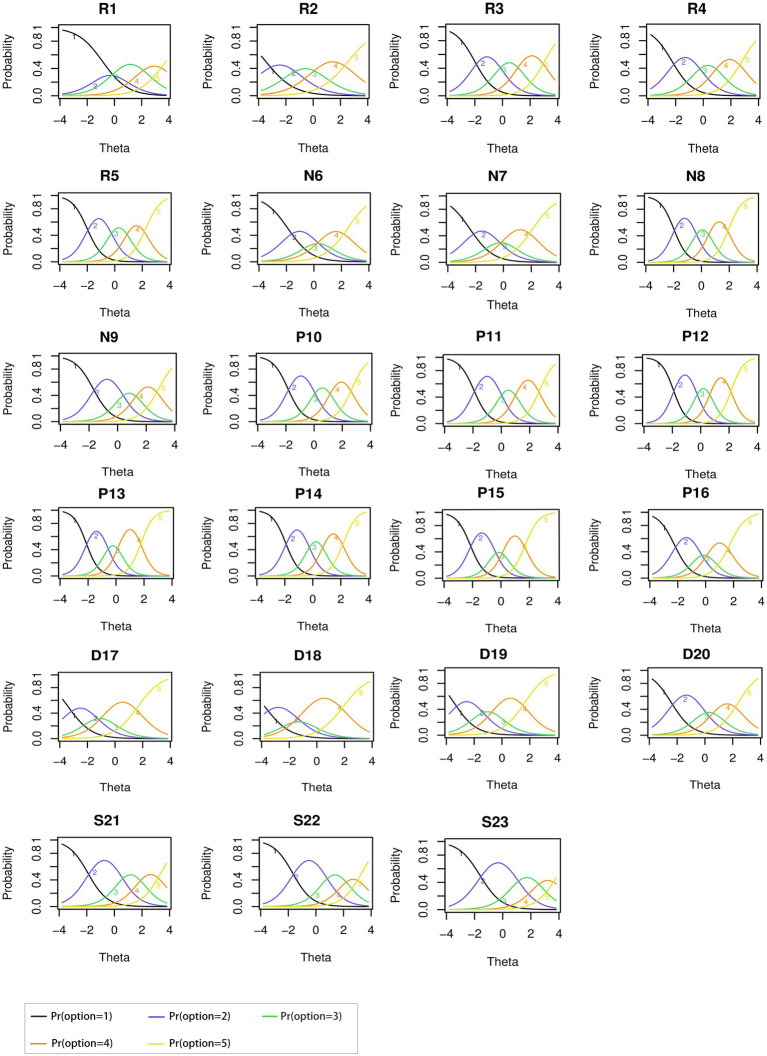
Item response category characteristic curves of all items in hepatitis B virus infection-related stigma scale (HBVISS).

Item response category characteristic curves depicted the relationship between the level of the patients’ perception of HBV-related stigma (i.e., the latent trait) and the probability of selecting the specific option for each item in the scale (Hendriks et al., 2012). The curves for all 23 items were shown in Figure 2, where the curves 1–5 in each plot corresponded to the five options (strongly disagree, disagree, neutral, agree, and strongly agree) of each item, respectively. Take item R3 as an example, patients with perceived HBV-related stigma (latent trait) levels below −2.1 were more likely to respond 1 (strongly disagree), while the patients with perceived HBV-related stigma levels between −2.1 and 0 were most likely to respond 2 (disagree). The curves of five options for all items showed the same character in-4 to 4 --curves 1 and 5 were monotonically decreasing and increasing respectively, and curves 2–4 were symmetrically distributed, with no overlap, indicating reasonable option settings and ideal measurement performance of all items (Figure 2).

#### 3.4.4. Information curve and IRT-based marginal reliability

**Figure 3:**
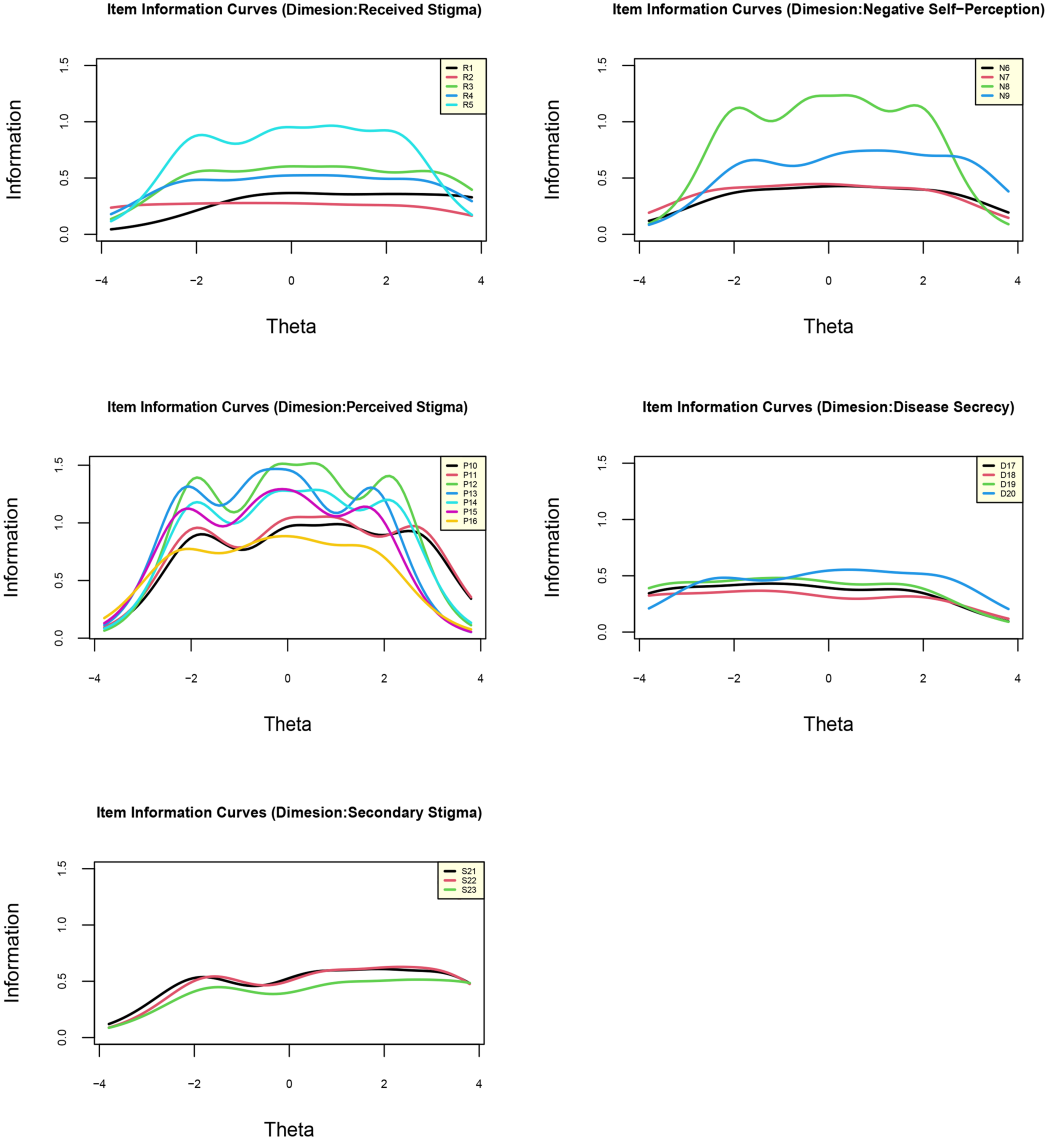
Item information curves of five dimensions of hepatitis B virus infection-related stigma scale (HBVISS).

**Figure 4:**
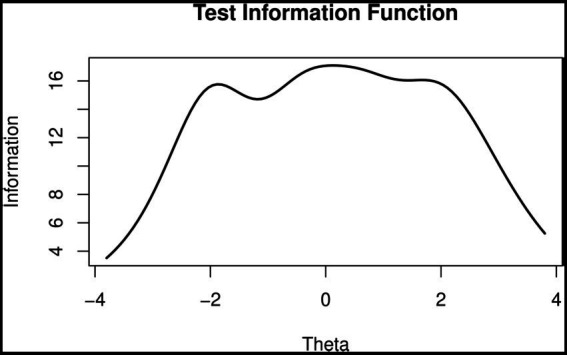
Test information curve of hepatitis B virus infection-related stigma scale (HBVISS).

Item information curves (Figure 3) showed that the items of “Perceived stigma” (P11-P15) possessed the highest information among the five dimensions, indicating the importance of the response of this specific dimension in terms of the measurement of perceived stigma level. Moreover, test information curves (Figure 4) showed that the 23 items altogether provided an abundant amount of information for measuring perceived stigma level ranging −2 ~ 2. Figure 4 also showed that when the stigma level equaled to 0, the test (or scale) information was the highest, highlighting the precision of the estimated results. The average test information that we calculated reached 12.75, proving that our scale could provide reliable results (information >10.0). The IRT-based marginal reliability was 0.95 for the total scale and 0.83–0.91 for the dimensions (Table 6).

**Table 6:** Marginal reliability of each dimension and total scale.

Dimensions	Marginal reliability
Received stigma	0.84
Negative self-perception	0.83
Perceived stigma	0.91
Disease secrecy	0.86
Secondary stigma	0.85
Total scale	0.95

#### 3.4.5. Differential item functioning

We did not detect DIF for gender, age, case source, residence, marital status, education, occupation, income, and diagnosis. The McFadden’s pseudo *R*^2^ value for change (d-*R*^2^) was <0.02 for all items, which means that the results of each item are not affected by the covariates. In terms of the DIF magnitude, the two highest values were detected when analyzing the age DIF for item R1 [I once suffered from unfair treatment because of the hepatitis B infection (rejected from admission, work, dating, marriage, sex, etc.)] (d-*R*^2^ = 0.019) and income DIF for the item D17 (“I do not want to tell others about my hepatitis B infection.”) (*d*-*R*^2^ = 0.016). However, the DIF magnitude was lower than the threshold d-*R*^2^ effect size of 0.02, indicating a negligible impact.

## 4. Discussion

To the best of our knowledge, this HBVISS is the first scale measuring HBV-related stigma from the perspective of patients in the local cultural context. This study validated the psychometric properties of HBVISS in a combination of CTT and IRT methods. Compared with the preliminary study on validity and reliability of the scale, our study not only had a larger sample size but also used more comprehensive analysis methods. Therefore, it is of great significance to the general application of HBVISS and may help collection of evidence for the advances of policies reducing the stigma against HBV infection.

As the results have shown, CTT analysis enabled us to have an overall grasp of the reliability and validity of HBVISS, while IRT showed us the characteristic of each item. The estimation of parameters and the calculation of information were also instrumental for the future development of computerized adaptive testing, so as to allow the medical care providers efficiently screen out the HBV-infected patients who perceived high levels of stigma and provide tailored nursing for their psychological health. In addition, comparisons of the item parameters and the exploration of the underlying reasons could direct us to develop the short form of scale. For example, item R2 had the lowest discriminative power among the 23 items. We think the possible reasons are as follows. On the one hand, as a review concluded, both individuals and their family members are prone to conceal the fact of HBV infection for the fear of discrimination from society (Lee et al., 2016). On the contrary, patients’ family members may also seek medical advice from others to support the patients. Hence, if an overall short form of HBVISS is to be constructed, item 2 may be removed from the short scale with minor accuracy compromise and remaining integrity of the measurement.

Different from other developed scales that focus on the views of the public toward HBV patients (Cotler et al., 2012; Shi et al., 2013), our scale is more intrinsic to patients’ inner feelings. Currently, HBVISS has been used in a few clinical research to investigate HBV patients’ perception of the stigma, so as to explore its mutual effect on social support, self-management behavior, and narrative nursing (Huang et al., 2021; Wang and Li, 2021a,b). To some extent, HBVISS would be of great significance to improving patients’ quality of life (QOL). Although QOL is still a subjective and vague concept, it is generally accepted that the individual is the most suitable judge of one’s own QOL (Hacker, 2010). In other words, physicians’ beliefs about the subjective states of their patients may be imprecise and inaccurate (Dorian and Brijmohan, 2020). Therefore, with our validated five-dimensional measurement tool, it is possible to quantitatively estimate the importance of specific interventions for patients’ QOL improvement and compare the effectiveness of different interventions from the self-assessed perspective of patients.

Several study limitations should be noted. Firstly, although we measured reliability using several different methods, the test–retest reliability of HBVISS was not evaluated. Secondly, this scale was developed in the Chinese cultural context and has been validated in Chinese. To be applied in diverse populations and different countries, further back-translation, culture adaptation, and pretesting are needed according to the guidelines (Beaton et al., 2000; Sousa and Rojjanasrirat, 2011). However, the strengths of the current study can still be noted. First, a large sample size of 1,058 patients completed the scale, making the results more reliable. Second, we used not only CTT but also IRT method to depict a precise characterization of the psychometric properties of each item, providing strong evidences for item validation and practical references for future application and interpretation of HBVISS. Last but not least, we provide evidence to support the psychometric properties of our scale, which is very limited among current HBV-related stigma instruments (Smith-Palmer et al., 2020).

## 5. Conclusion

Hepatitis B virus infection-related stigma scale is a 23-item instrument that can be used to measure chronic HBV infection-related stigma in China. Our analysis showed good feasibility, reliability, validity, and item quality (discrimination, threshold, information, and measurement invariance). Our results indicate that HBVISS is an appropriate tool for the assessment of chronic HBV infection-related stigma in the Chinese context.

## Data availability statement

The raw data supporting the conclusions of this article will be made available by the authors, without undue reservation.

## Ethics statement

The studies involving human participants were reviewed and approved by the Ethics Committee of School of Public Health, Sun Yat-sen University. The patients/participants provided their written informed consent to participate in this study.

## Author contributions

SZ: conceptualization, formal analysis, investigation, methodology, validation, visualization, and writing–original draft. YZ and WZ: formal analysis and methodology. LF: data collection. JG: survey arrangement. XL: conceptualization, funding acquisition, methodology, and project administration. YH: conceptualization, funding acquisition, methodology, and project administration. All authors contributed to the article and approved the submitted version.

## Funding

This study was supported by National Science and Technology Major Project of China, Grant Number 2018ZX10715004 and Guangdong Basic and Applied Basic Research Foundation, Grant Numbers 2020A1515011294, 2020A1515110230, 2021A1515011765, and 2021A1515011591. The sponsor of the study had no role in study design, data collection, data analysis, data interpretation, or writing of the manuscript.

## Conflict of interest

The authors declare that the research was conducted in the absence of any commercial or financial relationships that could be construed as a potential conflict of interest.

## Publisher’s note

All claims expressed in this article are solely those of the authors and do not necessarily represent those of their affiliated organizations, or those of the publisher, the editors and the reviewers. Any product that may be evaluated in this article, or claim that may be made by its manufacturer, is not guaranteed or endorsed by the publisher.

## Abbreviations

HBVISS: Hepatitis B Virus Infection-related Stigma Scale
CTT: Classical Test Theory
IRT: Item Response Theory
COSMIN: COnsensus-based Standards for the selection of health Measurement INstruments
PROMs: Patient-Reported Outcome Measures
DIF: differential item functioning
HBV: Hepatitis B virus
CHB: Chronic Hepatitis B
SD: Standard Deviation
CFA: confirmatory factor analysis
EFA: exploratory factor analysis
RMSEA: root mean square error of approximation
SRMR: standardized root mean square residual
GFI: goodness-of-fit index
CFI: comparative fit index
IFI: incremental fit index
TLI: Tucker-Lewis index
QOL: quality of life
